# Fear of making a mistake: a prominent cause of stress for COVID-19 ICU staff—a mixed-methods study

**DOI:** 10.1136/bmjoq-2022-002009

**Published:** 2023-01-25

**Authors:** Cecilia Escher, Elisabeth Nagy, Johan Creutzfeldt, Oili Dahl, Mini Ruiz, Mats Ericson, Walter Osika, Lisbet Meurling

**Affiliations:** 1 Clinical Sciences, Intervention and Technology (CLINTEC), Karolinska Institute, Stockholm, Sweden; 2 Department of Anesthesia and Intensive Care, Norrtälje Hospital, Stockholm, Sweden; 3 Center for Advanced Medical Simulation and Training (CAMST), Karolinska University Hospital, Stockholm, Sweden; 4 Department of Perioperative Medicine and Intensive Care, Karolinska University Hospital, Stockholm, Sweden; 5 Neurobiology, Care Sciences and Society, Karolinska Institute, Stockholm, Sweden; 6 Division of Ergonomics, Royal Institute of Technology, Stockholm, Sweden

**Keywords:** COVID-19, critical care, fatigue, safety culture

## Abstract

**Introduction:**

The COVID-19 pandemic has had a profound effect on many domains of healthcare. Even in high-income countries such as Sweden, the number of patients has vastly outnumbered the resources in affected areas, in particular during the first wave. Staff caring for patients with COVID-19 in intensive care units (ICUs) faced a very challenging situation that continued for months. This study aimed to describe burnout, safety climate and causes of stress among staff working in COVID-19 ICUs.

**Method:**

A survey was distributed to all staff working in ICUs treating patients with COVID-19 in five Swedish hospitals during 2020 and 2021. The numbers of respondents were 104 and 603, respectively. Prepandemic data including 172 respondents from 2018 served as baseline.

**Results:**

Staff exhaustion increased during the pandemic, but disengagement decreased compared with prepandemic levels (p<0.001). Background factors such as profession and work experience had no significant impact, but women scored higher in exhaustion. Total workload and working during both the first and second waves correlated positively to exhaustion, as did being regular ICU staff compared with temporary staff. Teamwork and safety climate remained unchanged compared with prepandemic levels.

Respondents reported ‘*making a mistake*’ as the most stressful of the predefined stressors. Qualitative analysis of open-ended questions identified ‘*lack of knowledge and large responsibility*’*, ‘workload and work environment*’*, ‘uncertainty*’*, ‘ethical stress*’ and ‘*organization and teamwork*’ as major causes of stress.

**Conclusion:**

Despite large workloads, disengagement at work was low in our sample, even compared with prepandemic levels. High levels of exhaustion were reported by the ICU staff who carried the largest workload. Multiple significant causes of stress were identified, with fear of making a mistake the most significant stressor.

**WHAT IS ALREADY KNOWN ON THIS TOPIC**

- Before the pandemic, fatigue and burnout were known as common problems among intensive care staff, and data have indicated negative effects on patient safety. The extreme situation during the first part of the COVID-19 pandemic calls for studies from an intensive care unit (ICU) staff perspective.

**WHAT THIS STUDY ADDS**

- ICU staff and non-ICU staff coming to help were under enormous pressure but still maintained a commitment to patient safety.
- Organisational factors such as short notice regarding working hours were a major cause for stress.

**HOW THIS STUDY MIGHT AFFECT RESEARCH, PRACTICE OR POLICY**

- Scheduling of shifts in advance is of importance to decrease staff stress. Departments must consider that regular ICU staff over time carries a heavier burden compared with staff coming to help. Departments and researchers should focus on strategies for managing high workload situations and helping ICU staff to recover.

## Introduction

### COVID-19 intensive care in the greater Stockholm area

The first patient with confirmed COVID-19, caused by the SARS-CoV-2, admitted to a Swedish intensive care unit (ICU) was reported on 6 March 2020. The Stockholm and Sörmland areas were the most severely affected in Scandinavia.

During spring 2020, over 250 patients with COVID-19 were simultaneously treated in the ICUs in the greater Stockholm and Sörmland areas, which normally staff 100 ICU beds. During the first 2 years up to 31 December 2021, a total of 11 241 care occasions for COVID-19 were recorded in this area, according to the Swedish Intensive Care Registry.^1^ There were periods with shortage of all kinds of resources such as personal protective equipment (PPE), drugs, ventilators, beds and staff. The first pandemic wave was followed by a calmer period before the second wave hit this area in November 2020.

### Staff situation in COVID-19 ICUs

The situation during the first COVID-19 wave was different from what most of the staff had ever experienced.^2–4^ To manage the situation, nursing assistants, registered nurses, physiotherapists and physicians from operating departments, anaesthesia and general wards, as well as healthcare students, private caregivers and even non-medical staff were recruited to help in the area, as in other European countries.^5^

Regular intensive care staff (physicians, registered nurses and nursing assistants) were scattered, caring for patients in hastily equipped facilities not designed for intensive care, assisted by non-ICU staff. The newcomers had a short introduction, usually 1–2 days. They worked either in their own profession if possible, or as a nursing assistant. Therefore, the regular ICU staff had responsibilities of both caring for several patients more than usual and supervising colleagues not used to intensive care.

Members of the heterogenous care teams changed frequently, and their communication was impaired due to the PPE. Shortage of staff led to more and longer shifts and rotation between night and day shifts was common. Efficient treatment of severely ill patients with COVID-19 was uncertain, so guidelines were updated weekly. Because no relatives were allowed to visit due to the risk of infection, new tasks were added, such as daily updates by phone.

### Staff health

High levels of stress and fatigue among doctors and nurses have earlier been discussed by scholars.^6–8^ Stress and burnout among doctors have even been called an epidemic,^9–13^ conferring considerable costs on individual and societal levels.^14^ Females’ higher levels of burnout have been described as due to work–home conflicts.^15 16^

More severe conditions such as post-traumatic stress disorder (PTSD) have been described among employees in surgery, obstetrics and emergency care. This has been found to correlate to sick leave and staff leaving their jobs or even changing career.^7 17 18^

Higher levels of PTSD and psychological distress were found in a review including studies of staff working in previous viral epidemics such as SARS and Middle East respiratory syndrome.^19^ Similar results were found in a sample of US nurses comparing those working with patients with COVID-19 and nurses treating other patient groups.^20^ Working more than 40 hours/week was a further risk factor. High burnout scores among staff treating patients with COVID-19 have been found in several recent studies.^2 21–24^

### Patient safety in the COVID-19 ICU

Prepandemic studies have established staff safety attitudes to reflect patient safety measures such as medical errors, morbidity and mortality.^25–27^ The complex relation between safety attitudes and staff well-being has been discussed.^28 29^ Some scholars have considered safety culture as an aspect of work environment for healthcare staff.^30^

Due to the pandemic, normal standards for intensive care could at times not be maintained. A large study from the USA concluded that in periods when hospitals received many patients with COVID-19 and the proportion of these patients was large, patient mortality increased.^31^ Further, such a situation when normal standards of care cannot be maintained can cause staff moral distress, burnout and poor safety culture.^2 21–23 30^

The main aim of this study was to explore how ICU staff, both regular and temporary, assessed fatigue and safety culture during the first part of the COVID-19 pandemic in five hospitals in the Stockholm and Sörmland areas. We also aimed to elucidate how background factors and exposure to work affected burnout and safety attitude scores. A further aim was to identify and understand prominent stressors in this situation.

## Methods

This descriptive mixed-methods study is based in part on measurements using validated instrument and subjective rating data (quantitative), and in part on textual answers on open-ended questions (qualitative). It was conducted in units treating patients with COVID-19 in need of intensive care in five hospitals in the Stockholm and Sörmland areas. These comprised one large university hospital, Karolinska University Hospital, located at two different sites (Solna and Huddinge), as well as one medium-sized (Mälarsjukhuset Eskilstuna) and two smaller hospitals (Nyköping and Norrtälje). In total, 13 separate COVID-19 ICU units were distributed at the five investigated hospitals.

Information about the study was clarified as participants opened the link to the questionnaire. In cases with questionnaires on paper, information was at the first page and participants signed before answering to any questions. The signature page was removed before analysis to ensure anonymity. All participants were informed that if the questions shed light on something they felt worrying and thus in need of professional support, they were offered such contact.

### Participants, settings and response rate

**Figure 1:**
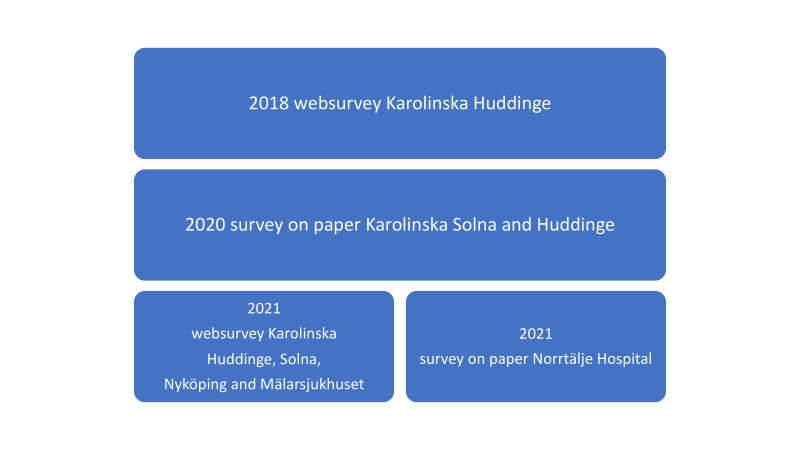
Data collection.

Data were obtained through surveys at three timepoints (figure 1).

The 2018 data were collected via a web survey as part of a teamwork training programme for registered nurses, nursing assistants and physicians at the operating department at Huddinge Hospital; the survey was distributed via email. These unpublished prepandemic data with 172 respondents served as background data. The response rate in this data set was 44%.

The 2020 data were collected during the last part of the first surge of COVID-19 in May and June. Printed questionnaires were placed in areas used by all ICU staff (physicians, registered nurses and nursing assistants) at Karolinska University Hospital in Solna and Huddinge. The anonymous questionnaires were collected in sealed envelopes or boxes. The total number of respondents was 104. The relative response rate for the 2020 data was not calculable due to the extreme situation the pandemic created and the unknown number of possible eligible participants among the different ICU staff.

The 2021 survey was a web survey, apart from in one of the small hospitals, where printed questionnaires were used (figure 1). Questionnaires were sent out by email to all regular and non-regular staff taking part in intensive care of patients with COVID-19 in the five hospitals since the start of the pandemic. The questionnaires were distributed on 8 March 2021, with reminders 10, 36 and 71 days thereafter.

A total of 1713 staff were invited, and 603 questionnaires were returned, giving a response rate of 35% (table 1). When trying to characterise the ‘non-responders’ only email addresses were possible to trace. An estimation based on 630 ‘non-responders’ revealed 74% females. No other analysis of the ‘non-responders’ was possible due to the anonymous design of the web tool used.

**Table 1:** Surveys and respondents

	2018	2020	2021
n	n=172	n=104	n=603
Female (%)	117 (68)	89 (86)	472 (78)
Age (years)			(n=603)
<40			213
41–50			198
>51			192
Age mean (years)	45	46	
Profession (%)			(n=603)
Registered nurses (RN)—all	79 (46)	72 (69)	344 (57)
RN specialist intensive care		36 (35)	180 (30)
RN specialist other (anaesthesia/OR)	80 (47)	26 (25)	133 (22)
Nurse assistant	42 (24)	13 (12)	159 (26)
Physician resident anaesthesia intensive care			17
Physician specialist anaesthesia intensive care		9 (9)	52 (9)
Physician—all	50 (29)	14 (13)	80 (13)
Other professions		5 (5)	20 (3)
Work experience (years)			(n=601)
<5			169
6–10			111
11–20			167
>21			154
Regular place of work			(n=602)
ICU (%)		41 (39)	291 (48)
Hospital*			(n=601)
Karolinska University Hospital Huddinge	172	78	212
Karolinska University Hospital Solna		26	190
Nyköping Hospital			73
Mälarsjukhuset Eskilstuna			87
Norrtälje Hospital			39
Volunteered to work in COVID-19 ICU			(n=601)
Yes (%)		50 (48)	337 (56)
Worked 12–13 hour shifts			(n=599)
Yes (%)		102 (98)	440 (73)
Crisis agreement†			(n=600)
Yes (%)			315 (52)

*42 staff worked in more than one of the hospitals in 2021; they were accounted to the hospital where they spent most time.

†Some hospitals made a special crisis agreement with employees that allowed the departments to schedule staff longer shifts and more than regular hours. It also enabled departments to demand staff to take extra shifts at short notice. The agreement included increased salaries.

ICU, intensive care unit; OR, operating room.

Posters were sent to the included hospitals in connection to the 2020 and 2021 surveys, describing the study and presenting contact information. Information was also given at staff meetings.

### Measurements

#### Background data and exposure

The participants answered questions regarding background factors including sex, age, profession, years in the profession and regular workplace.

Exposure to work in COVID-19 ICUs was characterised through questions on which out of the 13 COVID-19 ICUs they worked at, during which timeframe they worked, estimated number of working hours per week during the time in COVID-19 ICUs, typical shifts, working 12+ hour shifts, recruited to work on a voluntary basis to a COVID-19 and whether the respondent was included in a ‘crisis agreement’. (Some hospitals made a special crisis agreement with employees that allowed the department to schedule staff longer shifts and more than regular hours. It also enabled departments to demand staff to take extra shifts at short notice. The agreement included increased salaries.)

The questionnaire included the Oldenburg Burnout Inventory (OLBI), Safety Attitudes Questionnaire (SAQ) and questions about stressors.

#### Oldenburg Burnout Inventory

The OLBI is a validated instrument for assessing work-related stress and exhaustion.^32^ We used a Swedish translation, where the domains ‘disengagement’ and ‘exhaustion’ are self-assessed by the participants. The instrument entails 16 questions, eight for each domain, and is validated to Swedish circumstances.^33^ The answers are given on a Likert-type scale with 4 points from ‘strongly disagree’ to ‘strongly agree’.

#### Safety Attitudes Questionnaire

The SAQ (ICU version) is a validated instrument assessing healthcare workers’ perceptions of safety culture.^34^ The instrument was translated to Swedish in a previous study according to the International Society for Pharmacoeconomics and Outcomes Research guidelines.^35 36^ The SAQ includes six factors; of those, safety and teamwork climate were used. Respondents answer to the 13 questions on a 5-point Likert scale from ‘strongly disagree’ to ‘strongly agree’ or ‘not applicable’. Calculating groups of items provide the two factors of safety and teamwork climate. SAQ data are ordinal, though the usual method described, and used here, is to calculate and present the data as means.^34^

#### Stressors

Beyond the validated instruments, nine questions regarding perceived causes of stress at the COVID-19 ICU were asked on a 5-point Likert-like scale from ‘strongly disagree’ to ‘strongly agree’ or ‘not applicable’. The development of these questions was primarily theory-based knowledge concerning stressful factors known to be of importance in the ICU and modified according to the special circumstances of the expansion of intensive care during the pandemic, as well as on local discourse.^37–42^ Participants were also asked to add any other causes of stress they experienced in this setting.

### Analysis of qualitative data

The 2021 survey also included an open-ended question*: ‘What else caused stress in the Covid-19 ICU?*’. A total of 269 participants responded to this question. Thematic analysis, according to the principles described by Braun and Clarke, was used.^43^ The texts were coded and preliminary themes were found through an inductive process separately by two of the researchers. Preliminary subthemes and themes were identified, then negotiated and adjusted in an iterative process until consensus was obtained. The results were exemplified with quotes translated into English.

### Data management and statistical analysis

The data were summarised as mean and SD for continuous variables and number and proportion for categorical variables. A mean value was calculated for all questions regarding exhaustion and disengagement for each participant. All the negatively worded items were reversed before computing the mean values for all four outcomes.

To explore what factors were associated to the outcomes: exhaustion and disengagement from the OLBI instrument, teamwork and safety culture from the SAQ instrument and two linear regression models were computed separately for each of the four outcomes. The first model included respondents’ background factors; the second model included factors linked to exposure of COVID-19. Association between exhaustion and the different COVID-19 waves was investigated through regression mode including a categorical variable for wave. All models included hospital as a categorical explanatory variable.

Comparisons were conducted of the outcome disengagement, exhaustion, teamwork and safety between 2018 and 2020 as well as 2020 and 2021 using an unpaired unequal t-test to establish any significant differences between then mean value for all respondents between the 2 years. To control for size of hospital, data from the two university hospitals were also analysed separately.

To analyse which stressor scored highest (in terms of factor loading) on the questions on stress, structural equation modelling was used with stress as a continuous latent variable. The statistical analysis and data management were performed in STATA (StataCorp. 2017. Stata Statistical Software: Release 15. College Station, Texas: StataCorp).

### Patient and public involvement

The study population in this work is hospital staff. Development of research questions was done by the research group consisting of clinicians involved in clinical work in COVID-19 ICUs and external expertise. As clinicians we had close contact with our colleagues and were supported by them and heads of department. The predefined and open-ended questions regarding important causes of stress were derived from discussions and previous research.

Results from the study will be disseminated to, first, the departments and staff participating in the study and to the funder, AFA Försäkring. Our aim is that caregivers will participate in further dissemination.

## Results

### Quantitative data

#### Burnout scores (exhaustion and disengagement) 2018, 2020 and 2021

Compared with data collected in 2018 including physicians, anaesthesia and operating room registered nurses and nursing assistants working in one of the large hospitals in the study (n=172), exhaustion was significantly higher during the pandemic (table 2) in both the 2020 and 2021 surveys (mean 2.54, SD 0.55, p<0.001) compared with 2018 (mean 2.34, SD 0.53) surveys. On the other hand, disengagement was lower in 2020 and 2021 (mean 2.06, SD 0.57) compared with 2018 (mean 2.29, SD 0.31, p<0.001).

**Table 2:** Results comparing data from 2018, 2020 and 2021 (t-test)

Outcome	n	Mean (SD)	P value 2018	P value 2020
Ex 2018	172	2.3 (0.5)	–	<0.001
Ex 2020	103	2.7 (0.5)	<0.001	–
Ex 2021 (1/2)*	369	2.6 (0.6)	<0.001	ns
Ex 2021	551	2.5 (0.5)	<0.001	0.04
Dis 2018	172	2.3 (0.3)	–	0.005
Dis 2020	103	2.1 (0.5)	0.005	–
Dis 2021 (1/2)*	369	2.1 (0.6)	<0.001	ns
Dis 2021	551	2.1 (0.6)	<0.001	ns
SC 2018	172	59 (19)	–	0.015
SC 2020	103	54 (18)	0.015	–
SC 2021 (1/2)*	387	55 (19)	0.017	ns
SC 2021	574	59 (20)	ns	0.003
TC 2018	172	68 (18)	–	ns
TC 2020	103	64 (16)	ns	–
TC 2021 (1/2)*	385	68 (17)	ns	0.04
TC 2021	571	70 (17)	ns	<0.001

*The 2021 data including only the two larger hospitals (1/2).

Dis, disengagement; Ex, exhaustion; SC, safety climate; TC, teamwork climate.

#### Associations between background data, exposure to work and burnout outcomes 2021

In the regression analysis of background factors (table 3), female staff scored higher regarding exhaustion compared with men. No significant differences related to profession were found. Staff regularly working in the ICU scored higher regarding both exhaustion (p=0.014) and disengagement (p=0.012) compared with staff temporarily working in the ICU during the pandemic. Staff working voluntarily in the ICU scored lower in both exhaustion and disengagement (p<0.001). In the analysis of exposure to work in the COVID-19 ICU, exhaustion was higher in the group most exposed to work, that is, over 1628 hours (table 3). Likewise, working during both the first and second waves (p=0.012) was associated with exhaustion (table 3).

**Table 3:** Analysis of associations between background data and exposure to work and outcomes (regressions) 2021

	Exhaustion coefficient (95% CI) P value	Disengagement coefficient (95% CI) P value	Teamwork climate coefficient (95% CI) P value	Safety climate coefficient (95% CI) P value
**Background**				
Gender base Female				
Male	−0.16 (−0.27 to −0.04) 0.007	0.02 (−0.1 to 0.14) ns	2.5 (−0.86 to 5.9) ns	1.7 (−2.1 to 5.4) ns
Age (years) base 41–50				
18–40	−0.01 (−0.14 to 0.12) ns	0.13 (−0.005 to 0.26) ns	−0.23 (−3.9 to 3.5) ns	−2.6 (−6.7 to 1.6) ns
>51	−0.05 (−0.18 to 0.08) ns	0.001 (−0.14 to 0.14) ns	−0.41 (−4.2 to 3.4) ns	1.3 (−2.9 to 5.7) ns
Profession base RN				
RN specialist ICU	0.06 (−0.17 to 0.29) ns	−0.01 (−0.24 to 0.23) ns	−3.3 (−10.1 to 3.5) ns	−2.4 (−10.0 to 5.3) ns
RN specialist other	−0.04 (−0.026 to 0.18) ns	−0.03 (−0.26 to 0.19) ns	1.1 (−5.4 to 7.6) ns	0.76 (−6.5 to 8.0) ns
Nurse assistant	0.05 (−0.18 to 0.27) ns	−0.09 (−0.33 to 0.14) ns	0.89 (−5.6 to 7.5) ns	5.2 (−2.3 to 12.7) ns
Resident	0.06 (−0.28 to 0.40) ns	−0.17 (−0.53 to 0.18) ns	−3.9 (−14.1 to 6.2) ns	1.5(−9.9 to 12.9) ns
Specialist anaesthesia/ICU	0.05 (−0.21 to 0.31) ns	−0.22 (−0.49 to 0.05) ns	1.6 (−6.1 to 9.2) ns	9.4 (0.83 to 18) 0.032
Work experience (years) base <5				
6–10	−0.01 (−0.15 to 0.13) ns	0.03 (−0.11 to 0.18) ns	−5.0 (−9.1 to −0.87) 0.018	−1.4 (−6.0 to 3.2) ns
11–20	0.03 (−0.11 to 0.17) ns	0.09 (−0.06 to 0.23) ns	−4.5 (−8.6 to −0.51) 0.027	−2.9 (−7.4 to 1.6) ns
>21	−0.07 (−0.24 to 0.1) ns	0.02 (−0.16 to 0.19) ns	−0.9 (−5.8 to 4.0) ns	−0.03 (−5.6 to 5.5) ns
Regular workplace base ICU				
Non-ICU	−0.15 (−0.27 to −0.03) 0.014	−0.16 (−0.29 to −0.04) 0.012	4.9 (1.3 to 8.5) 0.007	0.70 (−3.3 to 4.7) ns
Hospital base Huddinge				
Solna	0.06 (−0.06 to 0.18) ns	0.005 (−0.12 to 0.13) ns	−0.84 (−4.3 to 2.7) ns	0.79 (−3.1 to 4.7) ns
Nyköping	−0.03 (−0.18 to 0.13) ns	−0.12 (−0.28 to 0.04) ns	6.5 (1.9 to 11.1) 0.005	14.6 (9.4 to 19.8) <0.001
Mälarsjukhuset Eskilstuna	−0.04 (−0.19 to 0.01) ns	−0.03 (−0.18 to 0.13) ns	6.9 (2.6 to 11.2) 0.002	10.0 (5.2 to 14.8) <0.001
Norrtälje	−0.21 (−0.4 to −0.02) 0.034	−0.31 (−0.51 to −0.11) 0.003	12.0 (6.3 to 17.8) <0.001	22 (15.5 to 28.4) <0.001
**Exposure**				
Voluntary base No				
Voluntary	−0.24 (−0.33 to −0.14) <0.001	−0.25 (−0.35 to −0.15) <0.001	6.7 (3.8 to 9.7) <0.001	9.1 (5.8 to 12.3) <0.001
12-hour shifts base No				
12-hour shifts	−0.01 (−0.17 to 0.15) ns	−0.06 (−0.23 to 0.11) ns	−0.9 (−5.0 to 4.9) ns	−1.4 (−7.0 to 4.1) ns
Crisis agreement base No				
Crisis agreement	0.07 (−0.04 to 0.17) ns	−0.02 (−0.14 to 0.09) ns	0.45 (−2.9 to 3.8) ns	1.8 (−1.9 to 5.5) ns
Workload (hours) base 925–1627				
<230	−0.31 (−0.49 to −0.13) 0.001	−0.30 (−0.49 to −0.11) 0.002	6.7 (1.2 to 12.2) 0.017	3.0 (−3.1 to 9.1) ns
231–480	−0.19 (−0.34 to −0.05) 0.01	−0.09 (−0.24 to 0.06) ns	−0.46 (−5.0 to 4.1) ns	−2.0 (−7.0 to 3.0) ns
481–924	−0.05 (−0.18 to 0.08) ns	0.03 (−0.11 to 0.16) ns	−0.19 (−4.2 to 3.8) ns	−1.3 (−5.8 to 3.2) ns
>1628	0.15 (0.03 to 0.28) 0.019	0.11 (−0.23 to 0.25) ns	−1.4 (−5.4 to 2.6) ns	0.2 (−6.7 to 6.5) ns
Work in relation to the 2 ‘waves’ base Not working during waves				
First wave	0.27 (−0.12 to 0.64) ns			
Second wave	0.10 (−0.30 to 0.50) ns			
Both waves	0.48 (0.11 to 0.86) 0.012			

ICU, intensive care unit; RN, registered nurse.

#### Associations between background data, exposure to work and patient safety attitudes 2021

Compared with data from 2018, safety and teamwork climate did not differ in the 2021 survey (table 2). Staff not regularly working in the ICU scored higher regarding teamwork climate (table 3). Scores regarding both teamwork climate (p<0.001 and p=0.005) and safety climate (p<0.001) were higher in the three smaller hospitals compared with the two larger hospitals (table 3). Physicians who specialised in anaesthesia and intensive care scored higher compared with nurses regarding safety climate (p=0.032).

#### Stress

Out of the nine stressors, ‘making a mistake’, ‘the short notice regarding working hours’ and ‘relatives cannot visit patients’ scored the highest (box 1).

Box 1 **Results scores of most important causes of stress**

- To make a mistake.
- The short notice regarding working hours.
- Relatives cannot visit patients.
- Not to find the equipment when needed.
- Not knowing in which ward I will work my next shift.
- Having insufficient knowledge regarding equipment or drugs.
- Not knowing my colleagues.
- To infect someone.
- To get infected myself.

### Qualitative data

The qualitative thematic analysis of answers to the open-ended question ‘What else caused stress at the Covid-19 ICU?’ resulted in five themes: lack of knowledge and large responsibility, workload and work environment, uncertainty, ethical stress, and organisation and teamwork.

#### Lack of knowledge and large responsibility

Both regular ICU staff and non-regulars expressed additional stress caused by the increased responsibility they had to take on, regarding both the number of patients to care for and task complexity. External staff were concerned about the short introduction, often 1–2 days, before caring for patients with COVID-19 in the ICU. ICU staff worried about the unfamiliar equipment, such as anaesthesia machines and old ventilators, which they had to use without proper or any introduction.

As a nurse anesthetist to be responsible for 2–3 Covid-19 ICU patients on different ventilators and try to manage the day together with an operating nurse and a nursing assistant. That is what it was like during spring 2020, we did not have any ICU staff. The anesthetists came and left but they were not there all the time.

The ICU staff expressed additional stress caused by a combination of very high workload and the worry that they could not support their non-ICU colleagues enough. Mentoring of new colleagues was expressed as a major stress factor among the ICU staff.

To be the one to lead the work besides doing my tasks was a challenge. The new colleagues, their fear for new, unknown tasks.

During the first part of the pandemic, lack of knowledge regarding the treatment of patients with COVID-19 was a concern. ICU staff were unfamiliar with the disease and expressed frustration both due to insufficient information and the rapid turnover of guidelines.

#### Workload and work environment

To staff the ICUs during periods with many patients with COVID-19, staff had to work longer and more frequent shifts. Due to the situation, with peaks in workload and high levels of sick leave among colleagues, staff also had to be available to take extra shifts at short notice. Both the large workload and the frequent changes of working hours were a major cause of stress. For some, this situation continued for many months, resulting in decreased time off duty and insufficient recovery time which was expressed as a stressor.

My private life was ruined as I did not have energy for social interaction, not even with my family. The short intervals between shifts were used for sleep and rest.

Because new COVID-19 ICU wards had to be located all over the hospital, not all were suitable for intensive care. Members of the staff were concerned about the risk of tripping on cables and equipment, as well as not having instant access to the patients or equipment when immediately needed. Larger rooms for 3–20 patients enabled staff to help each other but were very noisy.

The noise and many simultaneous alarms from patients.

Some wards had smaller rooms, but walls made them crowded and more difficult to survey.

In the x ward it was difficult to monitor three patients because of the walls. The y ward was very crowded, difficult to mobilize patients in rooms built for just one patient.

Personal protective gear was used in all COVID-19 ICUs, often over prolonged periods during long shifts. This meant limitations regarding possibilities to drink, eat and go the bathroom, which was expressed as a major cause of stress. The gear was also uncomfortable and warm; masks made it hard to communicate, verbally and visually, and at times they caused pain and wounds due to pressure.

Hard to communicate because of the gear. Difficult non-verbal communication like facial expression and atmosphere. Difficult to hear.

I did not recognize my colleagues in the personal protective equipment

#### Uncertainty

Insufficient deliveries of PPE, such as safe face masks, were concerns during the first wave. The risk of being infected at work was a worry because the paths for transmission of the virus were not yet fully understood.

Not knowing what kind of protection that is needed.

Running out of resources such as essential drugs, ventilators, beds and other equipment was a major concern. Not being able to take care of all patients in need and not being able to cope were expressed as stressors by the external but even more frequently by the regular ICU staff.

March to April 2020 we had a sense of panic when we ran out of all material, protective gear, disinfectant, drugs. A stressful feeling. We felt abandoned by the politicians

With time, COVID-19 ICU wards were more organised and new staff learnt essential skills. However, the workload was still very high on both external and ICU staff, and staff expressed being exhausted and distressed.

To be able to cope. Not knowing when it will end.

The high workload and long working hours. It was all new, non-voluntary, I felt exposed. I felt constantly tired, that made me not trust in myself, my judgement.

#### Ethical stress

Particularly during the first phase, normal standards for intensive care could not be maintained such as brushing teeth, changing patient position, changing syringes and close communication with the patient’s family. Not being able to meet ordinary standards was a great stressor for the ICU staff.

I did not have time for the compulsory care I know is essential for the patient. To risk missing the most important and ruin a patient’s life because of the stress.

I was very scared to miss a low blood glucose or potassium.

Due to risk of infection, no family members were allowed to visit the ICU wards. Both external and ICU staff expressed the stress of not being able to care for the dying and dead in a way that met their ethical standards.

Patients died alone.

Staff also expressed not being able to care for their colleagues as a stressor. Especially ICU staff often expressed seeing colleagues in distress but not having time to support or comfort them.

#### Organisation and teamwork

The external staff coming to help at the COVID-19 ICU were disconnected from their usual organisations and colleagues. They expressed a feeling of being abandoned because their managers were elsewhere with no superior in the ICU replacing them. Changing between different COVID-19 ICU wards enforced their feeling of being on their own to cope. The ICU staff were also stressed due to lack of continuity because the teams caring for one to three patients often changed from day to day.

To work in different wards each shift and the routines were not the same.

Some expressed cooperation between professionals as a source of stress.

Some colleagues don’t listen to my suggestions.

Doctors that have different opinions and repeatedly change the plan for the patients.

## Discussion

This mixed-methods study aimed to increase knowledge of the situation in COVID-19 ICUs from a staff perspective, including regular ICU physicians, registered nurses and nursing assistants, as well as staff coming to help from other units, in five Swedish hospitals. The main findings were increased burnout scores but stable safety attitudes compared with prepandemic data. We also identified several factors contributing to the stressful situation, with ‘making a mistake’ scoring the highest.

Burnout among doctors and nurses has been a problem in intensive care since before the COVID-19 pandemic. As expected, ICU staff in our sample scored highly in perceived exhaustion during the pandemic, significantly higher compared with our prepandemic data and displaying levels similar to other COVID-19 samples.^20 21 44^

Interestingly, our data displayed a decrease in disengagement during the pandemic. This finding has, to our knowledge, not been discussed by others. According to self-determination theory,^45^ external as well as internal factors are important for human behaviour and might provide an explanation. ICU staff were exposed to external motivators such as considerable public support and visibility in media during the pandemic, as well as internal motivators such as saving lives. We also found lower burnout scores among staff volunteering to work in COVID-19 ICUs, data that could be explained by even higher intrinsic motivation in the volunteer group. This is also in line with earlier research that showed social support^46^ and also perceived adequate reward buffer against disengagement and exhaustion.^47^

Burnout scores did not correlate to profession, age or work experience in our sample. A recent systematic review and meta-analysis of nurses’ burnout during the COVID-19 pandemic showed several associated risk factors such as younger age, working in a high-risk environment, increased workload and lower level of specialised training.^24^ Regarding workload, our study confirmed the correlation of burnout scores to total workload as well as working in both the first and second waves.

Women scored higher regarding burnout compared with men, and women also outnumbered men. These results are in line with earlier data suggesting work–home imbalance as a cause for burnout in females.^15 16 48^

Regular ICU nurses and physicians displayed higher burnout scores compared with non-regulars. To our knowledge, previous studies have not separately analysed regular ICU staff and non-regulars. Our qualitative data reveal the chaotic situation that staff faced during the first part of the pandemic. The regular ICU nurses and physicians had to take the responsibility to mentor colleagues, carry a large workload themselves and set aside standards of care. For the ICU staff, the double workload of many patients and mentoring new colleagues is a plausible reason for their greater exhaustion. Greater moral distress among the regular ICU staff could have contributed to their fatigue because they were aware of the normal ICU standards that had to be lowered during the pandemic.^3 23 49^

In our sample, safety and teamwork climate remained unchanged compared with our prepandemic data, and levels were similar to previous results.^35^ In a review, most but not all studies could establish a negative correlation between staff burnout and patient safety variables such as medical errors.^29^ Further, increased risk of patient safety events was found in a recent review on burnout among physicians.^50^

The staff-assessed safety climate remaining unchanged compared with prepandemic data in our sample was unexpected because burnout scores were high and ‘*the fear of making a mistake*’ was the number one cause of stress. A study from the UK also found puzzling patterns, but in that sample, professional groups displayed more diverse patterns.^51^

A review by Janes *et al* described a relationship between healthcare staff engagement and safety culture scores as well as errors and adverse events.^52^ One possible explanation to our findings could be that strong engagement among staff, at least in part, counterbalanced the negative effect of exhaustion on the assessed safety climate.

The number two cause of stress in the 2021 data was ‘*short notice regarding working hours*’. This result might reflect the loss of control affecting both private and work-life and is in line with a study on operating room staff’s perceptions of work during the pandemic.^53^

The number three cause of stress, ‘*relatives not being allowed to visit patients*’, seems to imply aspects of moral distress.^49^

In our qualitative data, staff reported separating severely sick patients from their loved ones as a major cause of stress. Clinical work is known to involve an emotional component, with the rewards of good relationships with patients and relatives normally balancing the work of caring for the distressed and dying. The COVID-19 situation has disrupted this balance.^54^ The combination of moral stress and increased workload in terms of working hours and task complexity has been a heavy burden for COVID-19 ICU staff. The moderate increase in exhaustion and low disengagement scores in our cohort can be explained by high motivation in this precarious time.

This study sheds light on the challenging situation assistants, nurses and physicians caring for patients with COVID-19 in intensive care faced during the first pandemic year. Our findings suggest that to maintain staff health in times of increased workload, scheduling shifts in good time is important. Keeping teams of staff together and avoiding changing wards should also be an aim in case of similar situations in the future. Identifying risk factors for burnout could be significant to allow healthcare staff and systems to respond better to epidemics and other circumstances involving increased workload in the future.

### Limitations of the study

Data from 2018 were collected among staff from one of the five included hospitals in the survey. The cohort in that sample was similar but not the same as the 2020 and 2021 samples. Nevertheless, analysis including only the one hospital did not change the significant increase in exhaustion or decrease in disengagement when comparing data collected in 2018 and 2021.

The 2020 data set was collected when staff was still working very long and frequent shifts, which may partly explain why the response rate was low. How the participants in the study differ from the non-responders is unknown. Most of the responders in the 2020 data set worked at the same hospital as the responders to the 2018 survey. Some differences exist regarding the background data, such as a larger proportion of physicians in the 2018 data set compared with 2021. Because profession did not correlate to any of our outcome measures, we consider this difference less important.

Information regarding staff choosing not to respond was limited in the 2021 data set due to the web-based data collection tool and the priority to ensure participants’ anonymity. The proportion of men/women, however, was similar. The response rate of 36% is a limitation but the level is similar to other studies. Parts of the research team were to some extent working in each participating hospital which might be looked on as a limitation.

When considering confounders to the result that men scored lower in exhaustion, we did not find any, such as profession or ICU as the regular workplace. Regarding the result that the three smaller hospitals scored better in some respects, we looked for confounders such as workload but found only minor differences in workload and working during the two waves. However, we had no access to any measures of staff to patient ratio or possible differences in patient characteristics that might have been important to workload. The difficulty to quantify the workload, not only in number of working hours per day or week or working during the waves of many patients at the ICU wards or not, makes the quantification of the total workload exposure almost impossible to estimate. Therefore, the study lacks the possibility to establish any truly reliable dose–response relationship, but the qualitative results presented give clues to what have been considered as important stressors for the clinicians at the studied ICUs.

## Data Availability

Data are available upon reasonable request. The qualitative data set is in Swedish language. The quantitative data can be shared upon request.
